# The Child’s Perspective on the School-Based Mindfulness Programme, Paws b

**DOI:** 10.1007/s10826-025-03047-6

**Published:** 2025-03-28

**Authors:** Katie Crompton, Alessandra Fasulo, Daphne Kaklamanou, Eszter Somogyi

**Affiliations:** Department of Psychology, Faculty of Health and Science, University of Portsmouth, Portsmouth, UK

**Keywords:** School-based mindfulness programme, Paws b, Primary-school children, Focus groups for children, Reflexive thematic analysis

## Abstract

School-based mindfulness programmes (SBMPs) are becoming widely used in primary schools, however findings regarding their effectiveness are controversial. Understanding how children describe and interpret the experience of taking part in these programmes may hold the key for improving their effectiveness. In this study we sought to gather children’s views about a 12-lesson SBMP called Paws b. A week after the completion of the SBMP in five classes of two primary schools, during Personal Social and Health Education (PSHE) lessons, we conducted four focus groups with 8- and 9-year-olds. We randomly selected two girls and two boys from each class to form each focus group. The discussion was led by a different researcher that had not been associated with the delivery of the lessons. A reflexive thematic analysis was conducted on the transcribed data. We identified three major themes in children’s discussions: (1) Mindfulness as instrumental for self-regulation, (2) Continued practice can lead to positive changes, and (3) Embedded memories from Paws b. The themes indicate that children remembered key practices and information, and used them in daily life. They enjoyed the training although not always from the beginning, observed changes in themselves and in their classmates and understood mechanisms through which mindfulness training can have positive effects. Implications of these findings are discussed in relation to both the content of this specific SBPM and the way in which the course was delivered.

## Highlights

- Focus groups were carried out with 8–9-year-olds to explore views and memories of the 12-lesson, school-based mindfulness programme (SMBP), Paws b.
- Children engaged in the focus group discussions with recollections about the teaching and examples from their and their classmates’ behaviour.
- Children reported using mindfulness practices to regulate negative emotions.
- Children explained how long-term mindfulness practice could facilitate positive change in behaviour.
- Children had had a positive experience with this SMBP and could name and demonstrate practices and knowledge gained from the course.

The field of mindfulness has experienced an exponential growth in publications, research and programmes in schools and universities over the past decade (Weare, 2019). The growth in school-based mindfulness programmes (SBMPs) has resulted in an expanding body of research examining the impacts of such programmes on student outcomes (Jennings, 2023). Empirical evidence of the beneficial effects of mindfulness includes improvements in children’s attention, well-being, self-regulation and cognitive control (Schonert-Reichl et al., 2015). Furthermore, there is promising evidence that these programmes reduce students’ feelings of anxiety and depression, support their physical health, and assist them in engaging in healthy relationships with others (Roeser et al., 2023). Mindfulness is commonly defined as “paying attention in a particular way: on purpose, in the present moment, and non-judgmentally” (Kabat-Zinn, 1994, p. 4). Mindfulness meditations involve selecting a point of focus, such as the breath, or a physical action such as raising and lowering arms, and regulating and directing attention to that point with sustained focused attention (Bishop et al., 2004). It enables those who practise it to be better able to be with their present experience, and respond more skilfully to what is actually happening in the present moment. (Costello and Lawler, 2014).

Most of the scientific literature on the benefits of mindfulness for children has focused on psychological and physical effects, however, there has more recently been a parallel interest in its inter-personal and collective effects (Kreplin et al., 2018). In a recent systematic review (Malin, 2023), SBMPs were considered in relation to how they may promote a positive school climate. Consistencies across studies were observed in emotional and behavioural regulation, prosocial behaviours, and reducing stress and anxiety in students. Findings suggest that SBMPs could be potential mediators in improving school and class climates. Quantitative studies have found an increase in certain prosocial behaviours following a school, or pre-school, mindfulness-based programme. Sharing behaviour increased in preschool children, following a kindness curriculum which had aspects of mindfulness within it (Flook et al., 2015). Following a 6-week mindfulness programme, preschoolers were rated as more prosocial by their teachers (Viglas & Perlman, 2018). Berti and Cigala (2020) found increases in observable sharing, helping and comforting behaviours during pre-schoolers’ play times, following a 6-week mindfulness intervention. Primary school aged children were rated as more prosocial by peers following a mindfulness intervention (Schonert-Reichl et al., 2015).

Malin (2023) suggests that these increases in prosocial behaviours develop through improved quality of relationships between students, their peers, and teachers. These findings have important implications for schools as a more positive class climate can have a direct effect on pupil and teacher mental health as well as academic achievement (Hamre & Pianta, 2001). Therefore, understanding how mindfulness may affect prosocial behaviour from the child’s perspective is important in gaining knowledge of the mechanisms which may be at play. One theory is that mindfulness practice may lead to increased empathy and compassion over time (Schindler & Friese, 2022). Cheang et al. (2019) examined the research to date which considered the link between mindfulness, empathy and compassion, involving children and adolescents. In their systematic review of quantitative studies, they found convincing support in favour of SBMPs increasing empathy in children and adolescents.

The majority of studies to date investigating SBMPs have focused primarily on quantitative measurements of programme outcomes (Dariotis et al., 2017), with less focus on in-depth explorations of individuals’ experiences of mindfulness, using qualitative methodologies. In particular, there has been minimal research examining how young children (five to 12 years old) perceive the practice (Ager et al., 2015). It has been suggested that there is a critical need to explore this growing field of educational practice by using qualitative or mixed methods research (Axford et al., 2022). Qualitative research is an important complement to outcome research on SBMPs, enabling participant-focused insight into the meaning and complexities of quantitative findings (Hutchinson et al., 2018). First-hand accounts from the children who participate in SBMPs, however, are rarely presented (Sapthiang et al., 2019). Understanding children’s perspectives on SBMPs can only serve to improve these programmes which are intended to benefit children themselves (D’Alessandro et al., 2022), through providing information about student learning and application of skills. This has potential to deepen our understanding of mindfulness mechanisms and guide future research and practice with respect to measuring proximal and distal outcomes of SBMP components (Dariotis et al., 2017).

Qualitative studies focusing on outcomes of SBMPs and yoga programmes have also described benefits with respect to social outcomes (e.g., self-assertion, improved peer relationships) (Case-Smith et al., 2010; Conboy et al., 2013). In their systematic review of qualitative research on children’s opinions of classroom-based mindfulness programmes, Sapthiang et al. (2019) highlighted four major themes, including using attentional processes to regulate emotions and cognitions, stress reduction, improved coping and social skills and calming and/or relaxation. Most qualitative studies have focused on high school-aged students however, with far fewer including elementary, or middle school-aged youths (Dariotis et al., 2017). One qualitative study (Ager et al., 2015), conducted in New Zealand, analysed 6–10-year-olds’ journals and focus groups throughout the SBMP, “Meditation Capsules” (Joyce et al., 2010). Student journals were recorded throughout the 10-week programme. Children wrote of increased awareness of themselves, others and the environment, and expressed that mindfulness would be effective to deal with conflicts with siblings and friends.

A United Kingdom (UK) based study (Thomas & Atkinson, 2017) investigated eight- and nine-years-olds’ and their teachers’ views on the same package used for this study, “Paws b” (Mindfulness in Schools Project; MiSP, 2023). This study investigated perceptions of Paws b specifically, rather than the concept of mindfulness in general, with a particular focus on how it could be instrumentally helpful in promoting attention and metacognition Thomas and Atkinson(2017) found that most pupils enjoyed the course, and it was deemed to be both accessible to pupils and feasible for teachers to deliver. Themes highlighted the impact of Paws b upon pupils’ attention, metacognition and social/emotional functioning, both within and beyond the classroom.

A second UK based focus group study of Paws b (Hutchinson et al., 2018) investigated how ten-eleven-year-old children who received long-term mindfulness training applied mindfulness to their everyday lives. The children in this study had received the Paws b course two years prior to the study and then for this study, reviewed the course and extended and embedded the learning into challenges that children may be facing, particularly regarding relationships, social media and transition experiences. The children continued to practise mindfulness in regular lessons across subjects where relevant and in an extra-curricular mindfulness club that met weekly as part of the embedding. Semi-structured interview questions explored the childrens’ experience of mindfulness and how they applied it to their lives. Four themes were identified: (1) processes of emotion regulation (2) dysregulation prompt to apply mindfulness (3) challenges and strategies and (4) the conditions that support or hinder mindfulness use. These themes revealed that mindfulness was assisting with children’s emotion regulation in times when they were experiencing difficult emotions.

The present study aims to investigate children’s understanding and application of mindfulness as well as their opinions of the SBMP, Paws b. It will be the first, to the best of our knowledge, to also investigate children’s perspectives regarding the possible link between mindfulness and prosocial behaviour. The study focuses on three research questions: What is children’s understanding of mindfulness; In what ways, if any, was mindfulness perceived by children to be instrumental in promoting prosocial behaviour and; What were children’s perceptions of the Paws b course? These research questions will be explored with children through the utilisation of focus groups. Focus groups allow pupils to express salient views; multiple voices can be heard and thus facilitate more rounded and reasoned responses to discussion questions (Barbour, 2007). They are also more economical than a series of pupil interviews, and are less intimidating to pupils (Cohen et. al., 2011).

## Method

### Participants

Participants were 15 eight- and nine-year-olds, from Year 3 and 4 children (boys 6; girls 9) from two state primary schools in Hampshire. These schools had a population with similar socioeconomic characteristics. Children were interviewed in four focus groups (Table 1). Pseudonyms were given for each of the children’s names.

**Table 1:** Participants in the four post SBMP focus groups

Focus Group	Pseudonyms (Age)
Group 1	Zander (8), Amy (8), Ollie (8), Milly (8)
Group 2	Joe (8), Harriet (8), Seb (8), Eden (8)
Group 3	Polly (8), Fred (8), Alice (9), Darcy (9)
Group 4	Sophie (9), Vicky (9), Carl (9)

### Procedure

Ethical approval was obtained from the Ethics Committee at the Department of Psychology at [removed for blind review]. An information sheet about the project was sent to all parents in Years 3 and 4, outlining the aims and methods of the research, and the benefits and risks involved. The only identified risk was the child missing thirty-minutes of their PSHE lesson time, however it was agreed by the teacher that there would be no need for the child to catch up on this missed lesson time. The Paws b curriculum was delivered to children in Years 3, 4 and 5. It was delivered to the children by the first author as part of her mixed methods PhD project investigating the effects of mindfulness on children. She is a trained Paws b teacher and also an experienced primary school teacher who primarily taught seven to nine-year-olds previous to the PhD. Focus groups in this study were planned to consist of four children in each group. Morgan et al., (2002) suggests that four or five participants are ideal, especially with younger children aged seven–eight years. Larger numbers with this age group can make it difficult for facilitators to encourage interactive discussion, and sessions can be noisy and therefore difficult to transcribe. Following completion of the SBMP, two boys and two girls were randomly selected from each of the Year 3 and 4 classes to take part in focus groups by entering all participant numbers into a random number generator computer programme. Consent forms were then sent to the parents of each selected child and if any declined or were not returned, another participant number was selected however due to child absences, two of the groups resulted with having three children in each. Participation in the focus groups was entirely voluntary for the children and required their verbal assent from the child on the day of the session.

The focus groups took place in the week following the final Paws b lesson; they were facilitated by Research Assistant (RA), who had facilitated a sharing task with all children involved, and so was familiar to the children, but had not been involved in delivering the mindfulness course. The RA had received training from the first and second author in how to carry out the semi-structured interviews with children. The first author had run a pilot focus group two days prior, in order to evaluate the questions and planned structure. This pilot focus group was audio recorded and shared with the RA and co-authors to help finalise the wording of the final questions.

In school A the focus groups took place in an out building known as “The Hut” and in school B they took place in a small classroom which was normally used by teaching assistants for interventions. Each group was interviewed for about half an hour using a semi-structured interview format. The focus groups were audio recorded and transcribed. A phone with the recording app “Voice Recorder” was placed in the middle of the table and turned on once the children had given verbal assent. Audio recordings were transcribed using the Dictate / Transcribe function in Word Online. This was completed by a trained RA and checked and edited by the first and last authors.

The first stage of the focus groups involved settling the children in and building trust with the interviewer, for example, by showing them the recording app on the phone, discussing confidentiality, and that their involvement was entirely voluntary. A warm-up activity invited children to draw a picture of one of the practices for other members of the group to guess the name of. The second stage was organised around a focus group protocol comprising interview questions (Table 2) that explored the experience of the SBMP from the child’s perspective. The interviewer asked each question exactly as it was worded on the protocol. She encouraged children to share ideas without putting their hands up to answer and responded with reflexivity within each focus group, allowing each child’s response to influence the next remark. For example, if a child said that they enjoyed the course, the interviewer may have followed up by asking them to describe what they enjoyed about it. Finally, the children were encouraged to write or draw a picture of something which came to mind, or was important to them, about mindfulness or the Paws b course and place it in an anonymous envelope. The aim of this final task was to capture any important perspectives that were less influenced by what children think adults want to hear.

**Table 2:** Focus group semi-structured interview protocol

Theme	Question
Understanding of Mindfulness:	“Remember the bubble we get ourselves into before we start a practice? I’m going to draw a big mindfulness bubble just like that on this piece of paper. Inside we can add all the words you can think of to do with mindfulness or Paws b. Which words come to mind when you think of mindfulness?” “If your friend asked you what mindfulness was, what would you say to them?”
Opinions of Paws b:	“When it was a Paws b day, how did you feel about doing a Paws b lesson?”
Usefulness of Mindfulness:	“When do you think someone might use mindfulness, or why might they use it?” “Have you ever used it? (why?)”
Prosocial Behaviour:	“This might sound like a funny question but do you think mindfulness could make a person more kind?” “How come?”

### The Paws b Course

Paws b is a course of six, one-hour long lessons, or twelve, 30-min lessons, offered formally as part of the Personal, Social and Health Education (PSHE) curriculum. It aims to teach seven-eleven-year-olds ways to regulate themselves when they are experiencing challenging feelings, how to relate to difficult thoughts and the story-telling mind, how to respond to difficulty rather than react, and ways of cultivating happiness. Children also learn about how key parts of their brain work, including the flight/fight/freeze stress response (MiSP (Mindfulness in Schools Project), 2023). The Paws b course is delivered with a mixture of didactic teaching using PowerPoint presentations, discussion and short experiential mindfulness practices. Home practice is encouraged but not compulsory (see Table 3 for a detailed description of the programme).

**Table 3:** Paws b lesson objectives

Lesson	Objectives
1	To introduce the idea of the mind and the brain as separate but connected To explore how the brain can be changed depending on how we train our minds To experience what it’s like to direct the attention To provide some simple tools for training the attention
2	To recognise that we have to make many choices in a day To understand that we can train our brain to be aware of when we make those choices To begin to train the mind in order to become more aware of our feelings and thoughts which can affect the choices we make Begin to recognise when there is an opportunity to make skilful choices
3	To introduce the idea of the faculty of attention To experience how we might direct our attention To understand the untrained mind’s fickle nature –it is like a puppy To learn some simple tools for training the attention with attitudes of kindness, patience and repetition
4	To introduce the idea of autopilot To explore how we can step out of autopilot when we choose to To understand the role of the hippocampus in connecting previous experiences with current ones To explore the everyday experience of stopping and ‘checking in’ with present moment awareness
5	Recognising that we all wobble Expanding breath awareness practices –finger breathing Exploring how to notice the wobble, and finding ways to steady ourselves Understanding how the Insula works with the Prefrontal Cortex to help us do this
6	Recognising how we notice when we (and others) are wobbling Learning how to steady ourselves when we notice the wobble Exploring settling attention in the lower part of the body as an anchor or steady base
7	Introducing the amygdala -learning to deal skilfully with difficulty Exploring the nature of mind and human patterns of reactivity Taking responsibility to keep the mind and body safe and healthy by choosing a response
8	Understanding when Fight/Flight/Freeze are important and when they are less helpful Exploring the difference between reacting and responding Practising pausing and choosing a different path Beginning to explore self-care and compassion –can we be kind to ourselves as well as others?
9	Exploring the nature of mind (trying to make sense of, filling in gaps, telling us stories) Learning to recognise thoughts (metacognitive awareness) Beginning to explore decentring from thoughts –thoughts are not facts
10	Understanding how thinking about what might be can exhaust us Learning to recognise how this can combine with body sensations, moods and actions Exploring how to use practice to steady and step back from difficult thoughts
11	Exploring how we can nurture ourselves and others Learning how to make room for and choosing happiness in our lives Noticing the details of experience of happiness Sharing happiness
12	Learning how to shift attention towards pleasant experience Understanding how savouring these experiences can increase levels of happiness Recognising the Paws b journey, and recalling what we have learned.

### Data Analysis

Data gathering resulted in four sets of children focus group data. The data set included anonymised transcriptions from the four focus groups together with the written words in the “mindfulness bubble” and any writing or drawings from the non-verbal, anonymous written task at the end. We utilised reflexive thematic analysis (Braun & Clarke, 2019) to identify, analyse and report themes developing from the data, conceptualising themes as patterns of shared meaning underpinned or united by a core concept, rather than themes as summaries of data domains (Braun & Clarke, 2019), or in our case, themes defined by our original research questions. By referring to Braun and Clarke’s (2013) 15-point checklist, throughout the analytical process, we were able to ensure a reliable and trustworthy analysis of the data. The themes generated from the data set are analytic outputs, developed through and from the creative labour of our coding. They reflect considerable analytic ‘work,’ and have been actively created by the first, second and last author, at the intersection of data, analytic process and subjectivity (Braun & Clarke, 2019).

Audio recordings of the data were listened to on multiple occasions and transcripts read and checked alongside the audio recordings for clarity. Coding was completed firstly by hand, on the transcripts themselves, and then transferred to spreadsheets where codes were grouped and categorised. The same data extract could have more than one code applied to it and each data item was given equal attention in the coding process. Codes represented the author’s interpretations of patterns of meaning across the dataset (Braun & Clarke, 2019). Only material that was irrelevant to the research questions was excluded, e.g. when the children diverted from a question to talk about the detail of a video game they played at home. When developing our themes, we endeavoured to stay closer to the children’s words while foregrounding the relevance of what they reported in sight of the research questions. Following Braun and Clarke (2019), this reflexive thematic analysis was a reflection of the authors’ interpretive analysis of the data conducted at the intersection of the dataset, the theoretical assumptions of the analysis, and the analytical skills/resources of the authors. Analysis was recursive, involving movement back and forth between the stages. At this stage the first author produced a hand-written mind map to collate the codes and data items relevant to their respective themes. The writing up process allowed for further editing and reviewing of themes and subthemes until they were felt to be internally coherent, consistent and distinctive. The transcripts and codes were reviewed by the second author, an experienced qualitative researcher, to enhance methodological rigour of the analysis. The themes were then reviewed against the original codes, by the last author to ensure, in-line with Braun and Clarke’s (2013) checklist, that the extracts illustrated the analytic claims. Results are presented as interactions between the interviewer and the participants. Each participant’s name is given, and responses are not chronological, but instead grouped according to how they best evidence each particular theme and subtheme.

## Results

The data provide a picture of children’s general perception of mindfulness and their opinions specifically relating to the Paws b course. We identified three major themes in children’s discussions: (1) Mindfulness as instrumental for self-regulation, (2) Continued practice can lead to positive changes, and (3) Embedded memories from Paws b. Subthemes for each of these themes are displayed in Table 4. All names provided in the quotes are pseudonyms, as presented previously, in Table 1.

**Table 4:** Themes and sub-themes arising from thematic analysis of the focus group data

Theme	Subtheme
1. Mindfulness as instrumental for self-regulation	Mindfulness as a tool to deal with negative emotions and difficult situations Mindfulness as a learning of behavioural techniques Mindfulness as a ‘calming’ tool
2. Continued practise can lead to positive changes	Mindfulness increases happiness Visible change in peers Bullies could become kinder
3. Embedded memories from Paws b	Enthusiasm about the course Areas of the brain Demonstrating practices

### Theme 1: Mindfulness is instrumental for self-regulation

The Mindfulness In School Project (MISP) provides, with the course resources, a presentation which can be used to introduce mindfulness to school staff. One slide of the presentation shows a picture of a toolbox, titled “The Mindfulness Toolbox” and is used to describe how practices can have a range of different positive outcomes, one of which is dealing with stress. Children are taught throughout the Paws b course that mindfulness practices can be used as a tool to help them in difficult situations, for example when they find themselves feeling stressed or worried. This learning of the instrumental application of mindfulness appears to have become embedded, with the identification of both the situations in which mindfulness can be helpful and the psychological effects of it. Subthemes were Mindfulness as a tool to deal with difficult situations and emotions, Mindfulness as the learning of behavioural techniques and Mindfulness as a “calming” tool.

Some children represented mindfulness as something they could resort to in particular situations in which they were feeling “frustrated”, “annoyed”, “stressed”, “sad” or “angry”. They evidenced this instrumental use of mindfulness by providing examples of when they had used it themselves and when and why others might choose to use a mindfulness practice. When asked when or why she thought someone might use mindfulness, Sophie answered:

If you feel frustrated with yourself of what you did and you just use one of those mindfulness practises like the tummy and chest or finger breathing one just to calm yourself down and feel like it’s okay, I’ve gotten over it, I can go onto the next chapter of my life now. (Sophie, July 2022)

Sophie’s words describe how, for her, mindfulness can release someone from a feeling of frustration and help them move on from it, rather than continually harbouring the unwanted feeling that is blocking her path. Similarly another participant, Darcy, describes how mindfulness can be used during a time when someone feels uncomfortable, and notes that it is possible to use a taught practice or make up a mindfulness practice. She suggests that the practice will help reduce or relieve uncomfortable feelings and instead provide more welcomed, “nice” feelings of relaxation and calmness, “If there was a time when you felt uncomfortable, you can - like- remember a mindfulness practice or you can make up one and it makes you feel quite relaxed and nice and calm.” The choice of phrase used by most children “calming down” is of interest as this was not a phrase used by the mindfulness teacher during the delivery of the course. It was a phrase, which children were familiar with, perhaps from hearing it from other adults in their environments. It is possible that the phrase “calm down” is a phrase the children hear from teachers and other school staff in an attempt to encourage self-regulation or reduce unwanted classroom behaviours such as aggression or over-excitement.

The children were taught that sometimes the amygdala reacts in certain situations, and by taking a step back and doing a mindfulness practice, the brain has a chance to respond, using the prefrontal cortex to make decisions, rather than reacting impulsively. This had become embedded learning for some of the children who talked about the Fight, Flight, Freeze (FFF) response. When discussing one of the videos they remembered from the course, Millie called out, “Oh, freeze, flight, no, fight, freeze,” to which Joe corrected, “Fight, flight and freeze.” The interviewer asked Joe what this means to which he replied, “So, fight is when you run away, freeze means you’re scared and you just stay still and fight is the one the baby did in the video”. He went on to explain how this was related to Paws b:

So, it’s *the amygdala* when you react and what they want to stop. I think it like just reacts and like doesn’t think about what it’s doing because it needs to do it really quickly. But sometimes it does it in not a very important moment when, like in a situation it does- it is not very important, but sometimes it does it and it’s really important, but sometimes it’s like no don’t need it, but it just does it. (Joe, July 2022)

A number of the children in the focus groups gave examples of situations where mindfulness could be instrumentally useful. These were often situations which could evoke a FFF reaction and could therefore be diffused by doing a mindfulness exercise. Vicky suggested that someone might use it, “when they are in an argument” and Carl added, “When they’re getting stressed or like put under pressure they might go away and do one.” Sophie went on to give an example of when she had used a mindfulness practice:

I did it in the car once because my dad, he can annoy me sometimes and we just on our way home from school and was like of by the way we are going round our friend’s house for a BBQ and I was like what, what? And I wanted to go home and just get dressed and get nice and clean…but then my dad said oh no it’s alright and so I got quite frustrated with my dad and I did one. (Sophie, July 2022)

There are hints here that mindfulness is used as a remedy, similar to how one may use medication, but to resolve an emotionally challenging scenario rather than a physical problem. When asked what mindfulness was, some children identified mindfulness as the course itself, describing the teaching and the techniques that were taught and others described the practices in the course. Joe explains,

mindfulness is where you have a teacher who every week on Fridays teaches you new courses and they go up to twelve, course twelve, and you learn how to do loads of things to calm yourself down when you get too excited, angry, when you can’t control yourself. (Joe, July 2022)

Millie adds:

So, it’s basically like where you have a teacher and then they teach you about like they teach you how to be calm when you’re going through a rough time and you’re getting upset or angry. And they give you one of those calming moves to help you control yourself, (Millie, July 2022)

to which Polly adds, “So, it’s kind of like learning but we do like exercises to calm ourselves down and we learn quite a lot of exercises.”

**Fig. 1:**
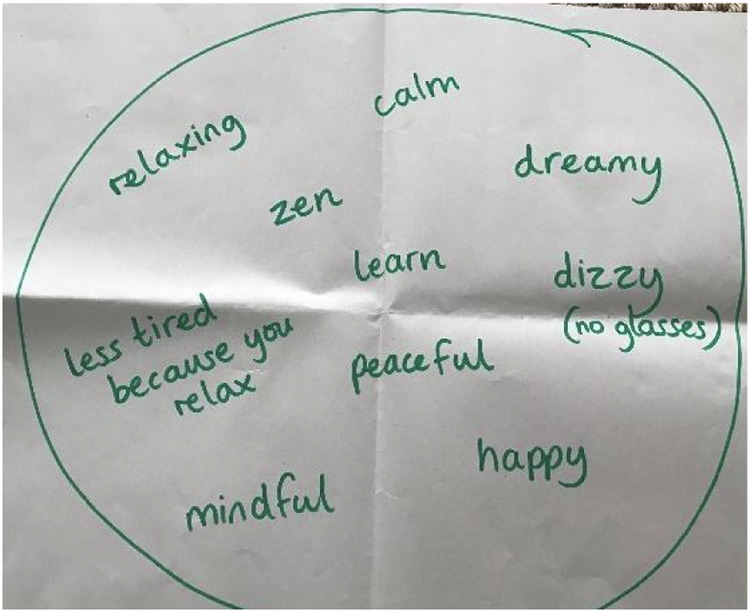
Group 3 Mindfulness Bubble

These responses show how children understand mindfulness as behavioural techniques which enable them to feel “calm”. The “calming moves” refer to the mindfulness practises the children are taught throughout the course. This result of feeling calm was not always in relation to resolving a difficult situation or emotion, it was just an observed outcome of practising mindfulness at any time. Vicky explains, “I like doing it because it makes me feel happy. It makes me feel calm.” Children were not taught explicitly that mindfulness is something which will make them calm down or feel calmer. In fact, during teacher training for the Paws b course, one of the learning points is that for many children mindfulness is not a calm experience and that for some, focusing on the breath in mindfulness practices can be challenging, for example if a child has asthma. “Calm” therefore was a child-initiated term which was used frequently in all of the four focus groups. For example, when children were asked in the focus groups to add words to a “mindfulness bubble” (a large circle drawn on a piece of paper) which came to mind when they thought of mindfulness, one of the most commonly used words was “calm” or variations of this word (e.g. calmness, calmfulness, being calm). Words with a similar meaning to calm such as “peaceful” and “relax” were also often suggested by children. Group 3’s mindfulness bubble shows a collection of their associations (see Fig. 1). Amber objectifies mindfulness as a calm and peaceful experience in itself describing it as “a calm and peaceful thing that just makes you feel relaxed.” Through children’s experience of learning mindfulness, they have come to their own analysis that mindfulness itself is about experiencing a state of calm and that the practices are peaceful experiences in themselves. “Calm down” is perhaps a phrase that children are familiar with hearing at school or at home. It may be a phrase that is used readily in schools by teachers and other school staff to remind children to be still, quiet, less active, more placid. [to be picked up in discussion about the situatedness of mindfulness teaching].

Paws b is aimed at providing children with tools, in the form of a number of practices, that they can use in times of difficulty. It aims to teach 7–11-year-olds ways to regulate themselves when they are experiencing challenging feelings, how to relate to difficult thoughts and the story-telling mind, how to respond to difficulty rather than react (MiSP (Mindfulness in Schools Project), 2023). This instrumental use of mindfulness seems to have been embedded for the children in the focus groups. Their description of it being a calm or calming experience, however, has developed through their own experience of practising mindfulness rather than being taught that this is what mindfulness will be.

### Theme 2: Continued Practise can Lead to Positive Changes

A number of children emphasised the effects of mindfulness on a different timescale to theme 1, reporting their understanding that mindfulness could facilitate long term positive changes both in how one feels and how one behaves. Children expressed the opinion that over the weeks, months and years a positive change could take place. They described how mindfulness leads to a happier life where a person is able to “notice the good stuff” and become more positive. Children described visible positive changes in the behaviour of certain individuals in their class and they suggested that bullies who were angry or mean could change and become kinder, through practising mindfulness. The children’s explanations and experiences of these long-term positive changes formed three subthemes: mindfulness increases happiness; visible changes in peers; bullies become kinder over time. Children suggested that practising mindfulness can lead to increased happiness in general. Carl says, “I like doing it because it makes me feel happy.”

One of the Paws b lessons, towards the end of the course, is about noticing happiness and focusing on the “good stuff”. Children had been taught earlier in the course that a person’s attention often shifts and that, through the development of their own practice, a person can direct their attention, as they wish. Children recognise, by the end of the course, that mindfulness is not only a tool to be used, for example, when they need to calm down, but that by practising mindfulness, long term increases in happiness are visible. Polly suggests, “I think people did mindfulness to have a happy life because even if there’s not something wrong it’s just good to do mindfulness because it actually makes you like more happy. That’s what I figured out.” This participant highlights that mindfulness does not only have to be used for self-regulation, to decrease unwanted or difficult feelings and emotions (as in theme 1), but that it can also be used to increase feelings of happiness. This increase in happiness was experienced by some participants through their own practice and for others, they noticed it in others who practised mindfulness. When asked if she thinks mindfulness could make a person kinder, Polly replied, “you probably are because I’ve noticed people are like more happy when they do mindfulness because if you’re in a positive attitude you can be more happy and you can do more good stuff.” This participant is explaining that she has noticed others who have been taught mindfulness are now happier. She explains it as them having a more positive attitude, allowing them to “do more good stuff”. She explains that she has seen a visible change in her peers in a positive way.

Children reported that mindfulness practice, over time, could lead to changes in attitude and behaviour, from negative attitudes and behaviours to more positive ones. Some children explained how they had seen a reduction of negative behaviours in their peers. This reduction in negative behaviours was visible in the form of less warning from the class teacher. Warnings are given to children when they display negative behaviours, for example, being aggressive to another child or choosing not to follow instructions from the class teacher. Zander acknowledged how a child in his class was receiving less warnings and mentioned this when asked if mindfulness could make a person kinder. “Some people might be getting kinder like Aaron. He was quite mean at the start of the year but now not getting as many warnings,” to which Ollie added, “Yeah, as we were doing it [mindfulness lessons] he started getting more calm and not getting much warnings now.” Millie was reminded of another child in the class who she felt had become calmer, “Tyler used to be really naughty and now he’s really calm. He doesn’t actually get any warning now.” Millie was asked by the interviewer if she thought this was from doing Paws.b. “Yeah, as we were doing it, he just started getting more calm and not getting much warning now.”

Children suggested that others who hadn’t been taught mindfulness and were angry or mean people, or bullies, could change and become kinder if they were to practise mindfulness over a prolonged period of time. There was agreement from most children that for these people, mindfulness does have the capacity to lead to increases in kindness. Carl said, “I kind of learned that bullies turn into bullies because they get bullied and if they learnt the mindfulness practice, they would really calm themselves down and probably forget about that moment and then be more kind.”

Children were able to explain how they saw mindfulness as a mechanism for increasing kindness through the development of calmness, as demonstrated in the quote above. Children expressed a belief that angry or mean children are not calm and so by teaching them mindfulness practices it will make them calmer and therefore kinder. They understood that this change in behaviour would take time and would not happen instantly, but that there would need to be repeated practice for a change to develop. Amy explained how mindfulness might make a person kinder: “When they are really angry, they get really mean but when they do like petal practice and finger practice, they might get more calm over the year. Over the year, the months and days they might get more kind.” And Joe added,

yes, because one reason people aren’t so kind is because they’re really active and then they’re really mean. But urm mindfulness is about being kind to others and then calming down. So, it kind of teaches you because you do it a lot. It’s a lot so it’s like teaching yourself to be kind, so I think it could make people who are mean kind. (Joe, July 2022)

Others reported that angry people are often unhappy with themselves or others, and described how mindfulness could make them kinder through the increase of happiness. Fred suggested that, “if you are angry then you can be a bit like unhappy with some people for no reason but if you’re mindful then you’re more kind.” And Amy agreed:

I think it does make you kind because like if you are like normally like not very happy like a down person then if you would like if something happened in your life and you got really upset about it and you never got happy. Then like if you’ve done one of the activities and just done some fun things in your life, maybe it makes you a bit happier and you just forget about all of it and then it makes you kind. (Amy, July 2022)

When asked this general question about prosocial behaviour, nearly all responses explained how a change could take place from negative behaviour to more positive behaviour. None of the children considered already kind children becoming kinder, but instead referred to children who were mean or angry and suggested how mindfulness could elicit a positive change in their behaviour.

### Theme 3: Embedded Memories From Paws b

When children were asked about their opinions of the Paws b course, there were certain elements of the course content and content delivery which appeared to be embedded in the children’s memories. These elements, which included learning about parts of the brain, the mindfulness practices, and videos demonstrating the Fight Flight Freeze (FFF) response, were recalled enthusiastically in all four focus groups. The children expressed positive responses about both the course content and the content delivery. They reported that they looked forward to the lessons and felt excited when they realised it was a Paws b day. During the focus groups children were forthcoming with knowledge they had gained throughout the course. They readily shared their acquired knowledge of parts of the brain, including the name, location and function of the four main areas they were taught about: the prefrontal cortex, amygdala, insula and hippocampus. Children voluntarily demonstrated mindfulness practices and offered instructions for how to perform them. Some were able to demonstrate the concept of mindfulness through their responses. Subthemes were: Enthusiasm about the course; Areas of the brain; Demonstrating mindfulness.

In general, when children were asked how they felt about the Paws b course, responses were positive and enthusiastic. Words such as “excited”, “happy”, “good” were often given. Children enjoyed the novelty of the course content. Carl said he felt, “happy because I like doing the exercises and we might learn about the parts of the brain and I quite like learning about the parts of the brain.” And Eden said, “I felt excited because I have never done anything like that before.” Sophie agreed, explaining:

When I wake up on Mondays knowing it’s a day when we’re gonna be learning about the brain, I get really excited because I really like learning about parts of the brain and how they affect your mind and things. (Sophie, July 2022)

They were particularly enthusiastic about the use of video clips to demonstrate key messages. Millie recalled the video “Because I’m Happy” which was played in the final lesson. She started singing the song during the focus group as she laughed at the memory of dancing around the classroom:

I was always quite excited because we usually watch a video. There was one where Logan was here for his last day with us and it was, we had a minion singing happy, it was like (sings some of song) then at the end of the day we just put it on because we liked it. (Millie, July 2022)

The course material appears to be in tune with what the children enjoy and find amusing and seems to have given children the means to embody and share positive emotions. This enjoyment of how the course content was delivered (through the use of amusing and entertaining videos) helped to embed these key messages and learning. When Joe was asked to think of any words to add to the mindfulness bubble, he suggested, “funny”. The interviewer asked him to describe what was funny and he said:

We saw a video of a person in the shop that wanted everything, literally all the candy, and then his dad said no, you’re not allowed that and he just went hyperactive and went to knock all that was put on the shelf. (Joe, July 2022)

Millie then added “Oh, freeze flight no fight freeze,” to which Joe corrected, “Fight, flight and freeze.”

There was a change in opinion from the beginning to the end of the course for a small number of children. These children explained that to begin with, they were unsure about the course and didn’t enjoy it. They talked about not knowing what to expect or thinking that all that “calm stuff” wasn’t for them. But these children explained that as they became familiar with the course content, they began to like it. When asked how he felt about doing a Paws b lesson, Joe said, “To start with I thought it was rubbish, then as got to know it, started to like it.” Zander added, “To start with it wasn’t really for me, it was all calm and stuff, then I got to experience it and I started to like it.” Seb then admitted his uncertainties to begin with, “I was scared at first but I just followed instructions and then I got into it.” This uncertainty seemed to be present, because mindfulness was a new concept which the children knew nothing about. They weren’t sure of what to expect from the lessons and the unknown made them feel uneasy. However, as the weeks progressed and they started to understand what the practices involved and what to expect from the lessons, their enthusiasm grew.

One aspect of the course which appeared to be deeply embedded in the children’s learning, and something they were keen to share with the interviewer, was their knowledge of different areas of the brain. Children could readily name the four areas of the brain which they had been taught throughout the course and many could also explain the function of each of these areas. Children were taught about the prefrontal cortex, the hippocampus, the insula and the amygdala. Ollie highlights this knowledge when he explains what mindfulness is:

Mindfulness is where you have a teacher who every week on a Friday teaches you new courses ….and you also learn bits about the brain, like the hippocampus, the prefrontal cortex. That’s what decides like everything. And then you’ve got the insula, amygdala, and that’s at the bottom of the hippocampus, there’s a little dot, and that’s called the insula, and its job is to connect memories to old memories. (Ollie, July 2022)

The children not only name the areas of the brain but offer information about where they are located and what their main role is. They talk enthusiastically about what they have learnt and enjoy discussing this knowledge amongst themselves in one of the focus groups. Millie says, “When she [the teacher] reminds us about the parts of your brain she goes, Okay shout it out, and we’re all like Prefrontal cortex, hippocampus, amygdala”. Zander adds, “Insula. I want to know the back of the brain; we only know the front of the brain.” At no point during the focus groups were children tested on their knowledge from the course or asked to share specific knowledge which they had learnt. Nevertheless, in each group there was a sense that children wanted to share their newly acquired expertise. When looking at the data, there was a vast amount of scientific knowledge about the brain which was readily shared. Children seemed proud that they knew four different parts of the brain, and were able to locate them and explain the job of each one.

**Fig. 2:**
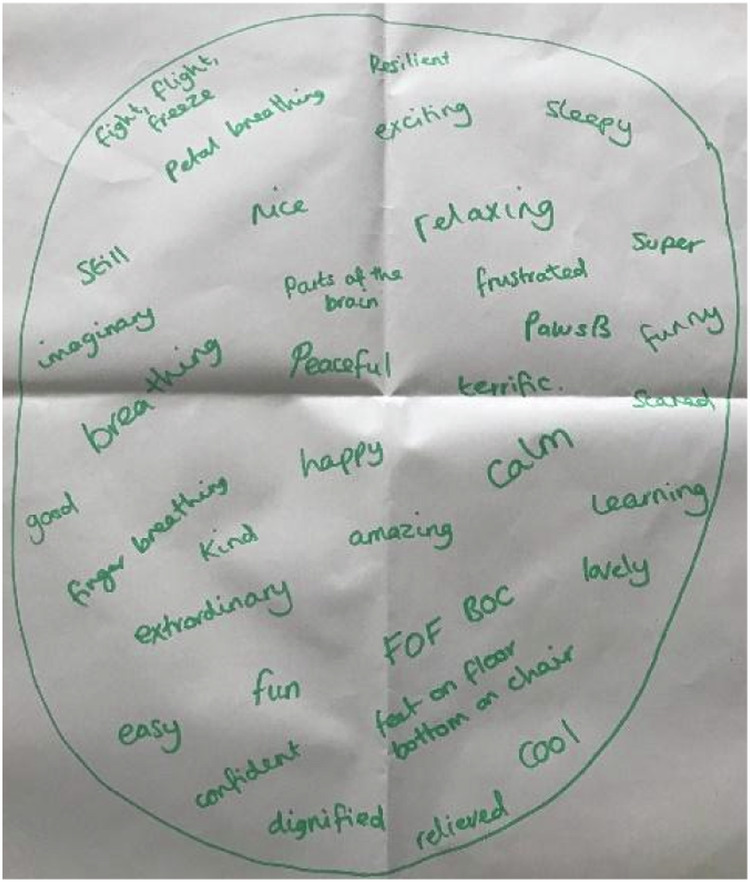
Group 2 Mindfulness Bubble

**Fig. 3:**
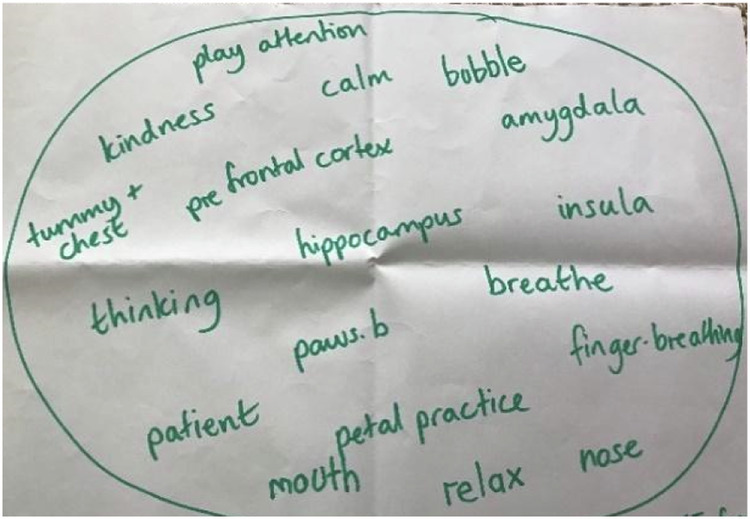
Group 4 Mindfulness bubble

A vast majority of the children in all of the focus groups were able to name the mindfulness practices they had been taught throughout the course. This was highlighted in the first task where children were asked what comes to mind when they think of mindfulness (see Fig. 2 and Fig. 3). The practices FOFBOC (feet on floor bottom on chair), Pause Be, Finger Breathing, Petal Practice and Tummy and Chest Breathing were all named in this activity. Millie was able to explain how to conduct the finger breathing practice. “Your fingers follow your breath” and Amy adds, “When you breathe out you go down and when you breathe in you go up. So, you’ve gotta try and get the same time as you breathe.” There were sections of the transcripts where children could be heard quietly breathing as they demonstrated “Petal Practice” and “Finger Breathing”. This learning seemed to be embedded through the aid of posters which were put up in each classroom as a reminder of the practices. Children noticed the posters and used them throughout the week. When drawing a picture of a mindfulness practice Milly explains, “We took them down last week. We had them up under our board.” Sophie adds:

The funny thing is that the one I’m thinking of, I wasn’t there for that one especially, but when I was in the line, I saw the poster and so I had a go at it and I liked this more than the ones I actually saw. (Sophie, July 2022)

Children chose to demonstrate their detailed understanding of the different mindfulness practices. Some children were able to offer explanations regarding mindfulness as a concept, referring to the ability to focus one’s attention on the present moment. They talked about the difference between being in a mindful state compared to that of being in a mindless state. When describing what mindfulness is, Alice says, “You don’t just do something whilst doing something else, you know what you are doing, you are aware of your surroundings.” Children were able to explain that a person’s attention can move around, by giving an example that if you touch or move a specific part of your body your attention will go to that area. They showed an understanding that a person can have control over where their attention is focused, by choosing to move or touch a certain part of the body. In the warm up activity children were asked to draw a practice, for others in the group to guess. One participant drew a practice called “Pause Be” and went on to explain how the practice works. In this explanation there was an understanding that a person’s attention can be pulled in many directions and that mindfulness is about noticing and choosing where and what to focus one’s attention on. Joe explains what a Pause Be practice is:

Stand up. So, you concentrate and you can wiggle your like toes if you are like trying to concentrate. Like basically look where your attention is, its where the part of your body’s most working. So, if you tap your head, your attention is on your head. It will be like wiggling our feet, no wiggling your toes so if you wiggle your toes your attention goes to your toes. And if you touch your head it might go to your head potentially. (Joe, July 2022)

In general children were enthusiastic about Paws b and readily shared their knowledge and understanding gained from the course. The delivery of the course content was suitably pitched to embed the learning. Children enjoyed the videos and when recalling them, the key messages were triggered, for example, the FFF response. The scientific element of the course, particularly learning about the brain, was popular, and possibly offered more concrete meaning to an otherwise novel and abstract concept. Nevertheless, children showed a sound understanding of the mindfulness practices and demonstrated them voluntarily during the focus groups.

## Discussion

To our knowledge this is the first qualitative study of children’s understanding of mindfulness and perspectives of the SMBP Paws b, with the additional focus on prosocial behaviour. Whereas most qualitative studies to date have focused largely on accessibility, feasibility and application of SBMPs, this study has provided information about children’s understanding of the concept of mindfulness and how it may affect behaviour from the child’s perspective. We have identified three major themes in children’s discussions: (1) Mindfulness is instrumental for self-regulation; (2) Continued practice can lead to positive changes; (3) Embedded memories from Paws b.

With regards to our first research question, “What were children’s understanding of mindfulness?” the data highlighted how children firstly view mindfulness as instrumental for self-regulation (Theme 1) and secondly describe continued mindfulness practice as leading to positive changes in behaviour. An exploratory study by Wisner, (2014) into children’s perceived benefits of mindfulness used concept mapping and organised responses into clusters. One of the clusters “more time spent being calm” included similar responses to Theme 1 (Mindfulness is instrumental for self-regulation), identified in this study. For example, in Wisner’s (2014) study children said “I felt calmer for the rest of the school day after meditation” and that “meditation helped me be calm in very intense situations”; Wisner suggests that mindfulness meditation may have facilitated intrapersonal changes through promotion of self-awareness, calmness, and improvement in stress management. There is support for these findings in the themes highlighted in this study, in that children explained how mindfulness makes them feel calmer and helps them deal with stressful situations.

Secondly, this study aimed to investigate, “In which ways, if any, mindfulness is perceived by children to be instrumental in promoting prosocial behaviour?” Children had noticed visible changes in some of their classmates, throughout the duration of the course. This included children being calmer and receiving less warnings from their class teacher for disruptive or unacceptable behaviour. They believed that mindfulness had the potential to decrease bullying behaviours, such as meanness, through the development of calmness and happiness. Children explained that this change in behaviour would be gradual, through continued practice over a period of time (Theme 2).

Similar to Sapthiang et al., (2019) who highlighted how children used attentional processes to regulate emotions and cognitions, much of what the children describe when they talk about how mindfulness can be used to calm down are elements of self-regulation. Self-regulation is defined as the deliberate use of skills to respond to demands of the environment in a contextually appropriate way and to achieve desired goals (Montroy et al., 2014). The children in this study report using mindfulness in times when they feel stressed, frustrated, angry or sad. They give examples of situations which could lead to these feelings, for example, arguments with friends or disagreements with family members. In their study, Montroy and colleagues suggest that self-regulation may be important not just because of the way that it relates directly to academic achievement but also because of the ways in which it promotes or inhibits children’s interactions with others. Children describe how mindfulness can be a tool for self-regulation and can thus change their behaviour towards others and improve interactions through acting in a kinder way towards them. This echoes Schindler and Friese’s (2022) suggestion that increased mindfulness is linked to improved self-regulation abilities, a mechanism for increasing prosocial behaviour. Children demonstrate their understanding of this mechanism when they explain that mindfulness can make you feel calmer which can make you kinder.

One alternative theoretical suggestion for how mindfulness may lead to prosocial behaviour is through increased empathic concern (Schindler & Friese, 2022). For the children in this study who thought that mindfulness could make someone kinder, there was nothing said by the children which could have been linked to this theory. Children did not talk about noticing how their friends were feeling, or anything else which could have suggested them being more sensitive to others emotional states. All of the children talked about mindfulness making a person calmer (self-regulation) or happier and this in turn leading to the potential increase in prosocial behaviour (specifically kindness). Self-regulation had more emphasis than empathy in children’s views.

Finally, the data enabled us to gain an understanding of children’s views on Paws b, linking to our third research question, “What were children’s opinions about the Paws b course?” Children shared fond memories of the course, with particular reference to the funny videos and clips teaching them about the FFF reactions (Theme 3). Although there was generally a very positive retrospective opinion of the course, some children report feeling negatively about the course to begin with. As the course progressed however, and they became more familiar with the course content and knew what to expect of the lessons, these children’s opinions changed and they began to enjoy it. (Theme 3). Children were able to name and demonstrate the mindfulness practices they had been taught and some children explained the difference between being in a mindful vs mindless state and how attention and focus can be directed to different parts of the body (Theme 3).

There were similarities between themes in this study and themes in other qualitative studies of children’s perspectives of mindfulness. Cruchon (2009, p. 50) found that “mindfulness/relaxation have the potential of helping young children feel more relaxed, less afraid, less tense, less sad and more happy.” These findings are closely linked to subthemes in this study: “Mindfulness as a calming tool” and; “Mindfulness increases happiness”. Although Cruchon used a combination of mindfulness and yoga, it appears that similar feelings of happiness and calmness are being expressed by participants. In our study however, there is a novel content of mindfulness techniques being seen as tools to be used in difficult moments or to overcome moments of tensions with other people. Furthermore, children go beyond the immediate effects reporting an understanding that mindfulness practices produce long term positive changes.

The focus groups highlighted how children perceive negative emotions as obstacles to positive relations. They see mindfulness as something that allows them to leave things behind more easily. They offered examples, based on their own experience, of how mindfulness has helped them do this. As for the thematic analysis of the qualitative data generated by the focus groups with children, dependability was high due to the specific use of Braun and Clarke’s (2012) six phase thematic analysis, which is highly prescriptive (Thomas & Atkinson, 2017). Using different methods of generating data from children during the focus groups (words children wrote in the “mindfulness bubble,” transcripts of the discussion and the non-verbal, anonymous task) was a form of ‘triangulation’ (using data of different types) (Hutchinson et al., 2018), and it was effective in that it enabled children with different preferences to illustrate their ideas the way that best suited them. For example, some children offered numerous verbal explanations, whereas others offered little verbally but instead offered opinions in the non-verbal, anonymous task.

Although the semi-structured focus groups provided rich, anecdotal data on children’s experiences of a SMBP, these data must be interpreted with caution as the perspectives shared in discussions were particular to Paws b and may not translate entirely to other SBMPs. Further research could explore findings from a larger variety of schools or locations. These findings cannot be generalised to all age groups and developmental differences must be considered.. Therefore, future research should consider the perspectives of both older and younger year groups in reference to SBMPs. There was also a lack of racial diversity across participants as the school populations themselves were not representative of areas with greater ethnic diversity. Replication in a more diverse sample could yield additional findings. Separate focus groups with parents or teachers may have further developed understanding around how children apply mindfulness in their lives. It would be interesting to investigate, for example, the impact of teachers’ understanding or opinions of mindfulness, on children’s mindfulness practice.

Collecting data from children poses a number of potential difficulties. A key task for the facilitator of focus groups is to maintain an appropriate balance of power in terms of directing and controlling the group, and creating an atmosphere in which participants feel free to discuss. This task poses a greater challenge with children, in view of the inherent power imbalance and the tendency to view the facilitator as an authority figure, such as a teacher, and respond accordingly (Morgan et al., 2002). It was important, for this reason, to provide children with the warm-up activity, where the RA was able to demonstrate an open and friendly demeanour, to help children feel at ease during the focus group. Secondly, the requirement to ask meaningful questions that will elicit detailed and relevant responses is particularly difficult in relation to children, given the differing ideas, understandings and social worlds of children and adults (Morgan et al., 2002). Having an awareness of this in our study meant that the RA could ask children to explain a little bit more about what they meant when necessary. One way of eliminating this in future research could be to record children’s discussions about mindfulness, in their own peer groups, without the presence of an adult facilitator asking questions.

Although the children shared positive feedback about the Paws b course, they were not asked about specific likes and dislikes or about how Paws b could be improved. In one sense, the open-ended question, “How did you feel on a Paws b day, about the Paws b lessons?” gave the chance for children to offer positive and negative feedback, however they may have given overly positive responses if they thought that would be favourable to the interviewer. Every effort was made to address this by having an RA interview the children, not the person who delivered the SBMP. Similarly, when children were asked if mindfulness could make a person kinder, demand characteristics may have led children to respond with what they thought the favourable answer would be. Nevertheless, children who thought mindfulness could make a person kinder were able to justify this by giving examples and sharing ideas about how and why this might be the case.

Finally, the results will have been influenced by the researcher’s own epistemologies, not least as a developmental psychologist but also as a former primary school teacher and a mindfulness teacher. Despite these potential limitations, the present study provides important results adding to the sparsity of qualitative studies investigating children’s perspectives on SBMPs.

## Conclusions

The aim of this qualitative study was to establish children’s views about a 12-lesson, SBMP called Paws b. Using focus groups to collect data from children, this study adopted an open-ended approach by considering children’s general perspectives of the SBMP, as well as asking them about its links to prosocial behaviour.

We identified three major themes in children’s discussions: (1) Mindfulness is instrumental in self-regulation; (2) Continued practice leads to positive changes; (3) Embedded memories from Paws b. Using these themes, we were able to conclude that, from the children’s perspective, mindfulness is a series of calming practices which can change children’s negative behaviours, to more positive behaviours, through increases in calmness or happiness and that long term mindfulness practice led to increased happiness in general. Children remembered key practices and information, and used them in daily life, expressing enthusiasm about the course content and content delivery. Further research is required to investigate how Paws b compares to other SBMPs and whether similar themes emerge.
